# Deep Semi-Supervised Algorithm for Learning Cluster-Oriented Representations of Medical Images Using Partially Observable DICOM Tags and Images

**DOI:** 10.3390/diagnostics11101920

**Published:** 2021-10-17

**Authors:** Teo Manojlović, Ivan Štajduhar

**Affiliations:** 1 Department of Computer Engineering, Faculty of Engineering, University of Rijeka, Vukovarska 58, 51000 Rijeka, Croatia; tmanojlovic@riteh.hr; 2 Center for Artificial Intelligence and Cybersecurity, University of Rijeka, Radmile Matejčić 2, 51000 Rijeka, Croatia

**Keywords:** deep clustering, semi-supervised learning, autoencoder, medical imaging, PACS, DICOM

## Abstract

The task of automatically extracting large homogeneous datasets of medical images based on detailed criteria and/or semantic similarity can be challenging because the acquisition and storage of medical images in clinical practice is not fully standardised and can be prone to errors, which are often made unintentionally by medical professionals during manual input. In this paper, we propose an algorithm for learning cluster-oriented representations of medical images by fusing images with partially observable DICOM tags. Pairwise relations are modelled by thresholding the *Gower* distance measure which is calculated using eight DICOM tags. We trained the models using 30,000 images, and we tested them using a disjoint test set consisting of 8000 images, gathered retrospectively from the PACS repository of the Clinical Hospital Centre Rijeka in 2017. We compare our method against the standard and deep unsupervised clustering algorithms, as well as the popular semi-supervised algorithms combined with the most commonly used feature descriptors. Our model achieves an NMI score of 0.584 with respect to the anatomic region, and an NMI score of 0.793 with respect to the modality. The results suggest that DICOM data can be used to generate pairwise constraints that can help improve medical images clustering, even when using only a small number of constraints.

## 1. Introduction

In the last few decades, medical imaging became a standard for non-invasive examination of the patient’s body interior in the clinic. To address the issue of storing and accessing medical images in a standardised way, PACS (Picture Archiving and Communication System) technology was developed to provide efficient and convenient data management, such that would make the images, among other things, easily searchable and retrievable. To make all medical images easily transferable between PACS repositories of different clinical centres, simultaneously providing interoperability between various medical devices, DICOM (Digital Imaging and Communications in Medicine) standard was developed, defining a format for storing images along with their related information that can be filled in manually by a medical professional or automatically by a device [1]. DICOM standard is available on the URL https://www.dicomstandard.org/ (last accessed on 1 October 2021).

Due to the not-so-infrequent changes in medical nomenclature and work routines that are often unique for specific individuals, as well as workload issues concerning the everyday engagement of medical professionals, the DICOM tags associated with medical images can be incomplete, erroneous, or even missing. Because of that, searching and retrieving similar clinical cases from PACS repositories, using DICOM tags, can be challenging [2].

**Figure 1:**
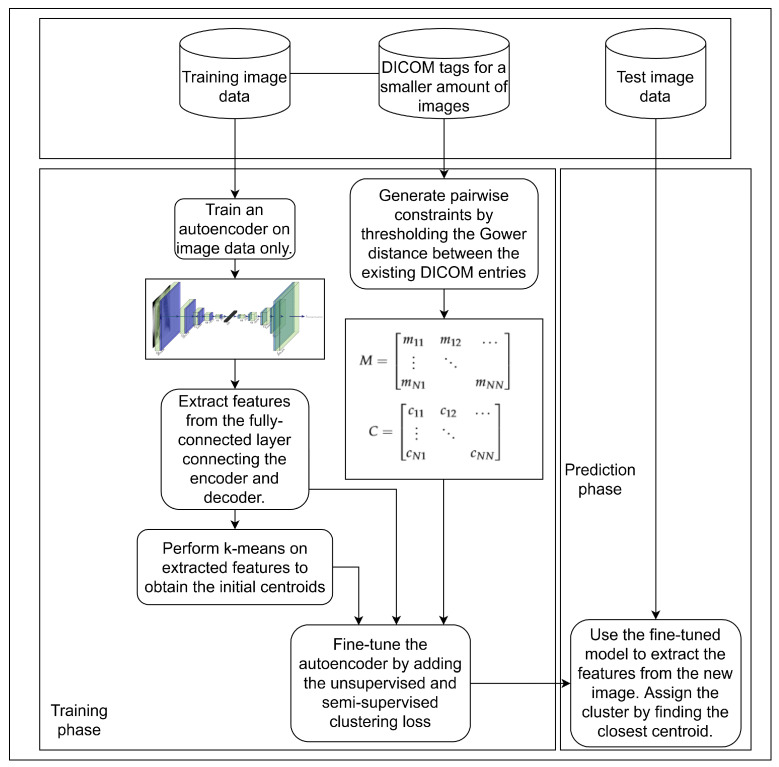
A flowchart showing the steps in the algorithm training and prediction phases.

Since the information tied to the images is sometimes known, e.g., when specific DICOM tag values are available, we hypothesise that constructing pairwise constraints based on available meta-information could improve the clustering result over those instances where the data is only partially available. Thus, we propose a semi-supervised clustering algorithm that utilises a sizeable collection of unlabelled data, consisting of images only, and a smaller amount of labelled data, consisting of images coupled with partially complete DICOM tags. Our algorithm consists of two steps. In the first step, we train a convolutional autoencoder (CAE) on images, only to obtain initial cluster embeddings which are then clustered using the k-means algorithm [3] to obtain initial cluster labels, as well as cluster centres. In the second step, we fine-tune our model using pairwise constraints, which are calculated from the DICOM tags, coupled with images to obtain a cluster-oriented latent space, enhancing model performance. The algorithm flowchart is shown in Figure 1.

The contributions of this paper are as follows:

- We propose a method for exploiting DICOM tag information to construct pairwise relations using the *Gower* distance. After the *Gower* distance is calculated, thresholding is applied to create *must-link* and *cannot-link* pairwise constraints. By using this distance, we address the issue of missing data as well as the heterogeneity of data types across features.
- Our method is not limited to data having a single target value. Instead, it can be used on data where each image can be described using multiple target variables, i.e., DICOM tags.
- To introduce pairwise information during training, we propose a cost function where, along with the classical deep embedded clustering (DEC) loss and the reconstruction loss, we minimise the Kullback–Leibler (KL) divergence between the distributions of instances belonging to the same cluster, while also maximising the KL divergence for the pairs not belonging to the same cluster.
- We compare our model against the unsupervised convolutional improved deep embedded clustering (IDEC) model and with the semi-supervised algorithms combined with the popular feature descriptors. Results show that using additional DICOM tags can improve the clustering performance.
- We show that the model generalises well by observing the two-dimensional t-SNE of the feature embedding space, calculated over a disjoint test set.

This work is structured as follows. In Section 2, we describe recently published work concerning the use and applications of DICOM tags as an information source, as well as current research concerning image clustering. In Section 3, we describe the proposed algorithm, the experimental setup, and the data used in the experiments. In Section 4, we describe the results and compare our model against similar models. Finally, in Section 5, we summarise and give directions for future work.

## 2. Related Work

Although the usage of DICOM tags in the categorisation of medical images is relatively unexplored, several papers dealt with this problem. Källman et al. [4] have shown that DICOM tags are useful in monitoring and optimising the patient radiation exposure index concerning medical imaging devices. This paper also reported that the acquisition of metadata can be done in a standardised way, irrespective of the PACS vendor, by constructing a workflow for periodical extraction and storing of DICOM images in a separate database—which can be then searched and processed using *structured query language* (SQL). DICOM data are relevant in PACS repositories where the search is carried out by using textual attributes; however, the format is not suitable for the web-based environment where most of the images are saved in JPEG (Joint Photographic Experts Group) or GIF (Graphics Interchange Format) formats [5]. Gueld et al. [6] used DICOM tags to perform medical image categorisation using four imaging modalities, achieving an error rate of 15.5%. However, we should note that the sample size used in this paper was relatively small. Manojlović et al. [7] compared the space of DICOM tags with the visual features of the medical images by clustering DICOM tags separately and observing how close the clustering results are in the visual embedding space. The presented results suggest there is a noticeable difference between the mean distance of cluster centres of images with those having cluster labels assigned by clustering DICOM tags, compared to those that were assigned randomly permuted cluster labels. Gauriau et al. [8] proposed a method for automating the identification of brain MRI sequences using metadata from DICOM tags, reporting the accuracy ranging from 97.4% to 99.96%, on a dataset of approximately 40,000 exams. Avishkar Misra et al. [9] used several DICOM tags to train a C4.5 model, having the goal of classifying lung regions, i.e., whether the region was apical, middle, or basal. They reported the lowest accuracy for the middle region (92.5%) and the highest accuracy for the apical region (96.6%). Although widely accepted by most medical-imaging systems manufacturers, we should note that the DICOM format does have some disadvantages. Lehmann et al. [10] characterised DICOM tags as roughly structured, ambiguous, and often optional. As an alternative, the authors proposed a mono-hierarchical multi-axial classification code format IRMA.

Because DICOM metadata was shown to be useful in several tasks, we hypothesise that some of the DICOM tags can be exploited for constructing pairwise relations which will improve the clustering results. Such algorithms that use small amounts of labelled data fall into the category of semi-supervised clustering algorithms, and there are numerous published papers where the performance of classical clustering algorithms, such as k-means, are improved using additional information. Two examples of such algorithms are constrained k-means (COP k-means) [11,12] and pairwise constrained k-means (PC k-means) [13]. However, when working with high dimensional and complex datasets—such as images, audio, or video—standard algorithms, e.g., k-means [3] or self-organising maps [14], on which the traditional semi-supervised clustering algorithms were based, perform poorly, mainly due to the inefficiency of the distance metrics used [15]. Sometimes, the data can even be too complex to be modelled using standard dimensionality reduction algorithms, such as principal component analysis (PCA) [16] or spectral methods [17]. To address those issues, researchers commonly use neural-network-based architectures to obtain a more feasible cluster-oriented data representation [15]. One such algorithm, namely DEC [18], was used in [19] to cluster the images from the PACS repository, outperforming the k-means algorithm with the most commonly used feature descriptors. There is also a report on utilising neural networks for generating pairwise constraints to improve clustering. In Hsu and Kira [20], pairwise constraints were utilised to learn cluster-oriented data representations by decreasing the KL divergence of the assignment probability for similar pairs while increasing the KL divergence of dissimilar ones. However, their approach does not involve using unlabelled data. Ren et al. [21] described a deep semi-supervised algorithm based on decreasing the *Euclidean* distance between pairs of instances that should be assigned into the same cluster while increasing the pairwise distance between instances that should not fall into the same cluster. In Tian et al. [22], a similar semi-supervised algorithm was proposed to analyse single-cell RNA-seq data, and compared with standard algorithms such as COP K-means and MPC K-means, showing a significant clustering improvement. Enguehard et al. [23] proposed a two-part neural network, consisting of a classifier part and a clustering part. However, in this approach, it is assumed that all labelled instances contain only one ground-truth label. Based on the assumption that binary classification is usually simpler than multi-class classification, in Śmieja et al. [24], a two-stage learning process was proposed. In the first stage, Siamese architecture was utilised to label pairs of data points to must-link or cannot-link. In the second stage, clustering was performed, having the highest reported NMI of 0.939 when using 5000 constraints. Zhang et al. [25] proposed a two-branch model for deep constrained clustering—where the first branch is used for instance-level losses (e.g., reconstruction loss, instance difficulty loss, or classical DEC loss), and the second branch is used to calculate pairwise losses. One epoch of the training of this model is performed firstly by iterating through all batches and updating the network using instance-level losses, and secondly, the network is updated using the pairwise constraints. Both Hsu and Kira [20] and Ren et al. [21] served as an inspiration for the model proposed in this paper. Our work is an extension and improvement of the work presented in [19], in which an unsupervised deep clustering algorithm was used to cluster medical images from a PACS repository.

## 3. Materials and Methods

In this paper, we propose an algorithm for learning semi-supervised clustering models which utilises two different types of data sources sequentially: (1) a larger number of unlabelled data consisting of images only, and (2) a smaller number of labelled data, consisting of medical images having at least one specific DICOM tag, required for constructing pairwise constraints. The method comprises several consecutive tasks, which are described as follows. First, in Section 3.1, we describe the CAE architecture which is trained on images and is used for calculating the first estimation of image embeddings. Next, in Section 3.2, we describe the *Gower* distance, which is used to construct pairwise relations from the DICOM tags. In this section, we also describe how pairwise relations are created and what type of relations can occur. Next, in Section 3.3, we describe a semi-supervised algorithm that utilises pairwise relations, as well as the images, to train the cluster-oriented embeddings. Finally, in Section 3.4 and Section 3.5, we describe the dataset that is used in this study, as well as the evaluation steps that were performed to evaluate algorithm performance.

### 3.1. Unsupervised Pretraining of a Feature Extractor on Images

**Figure 2:**
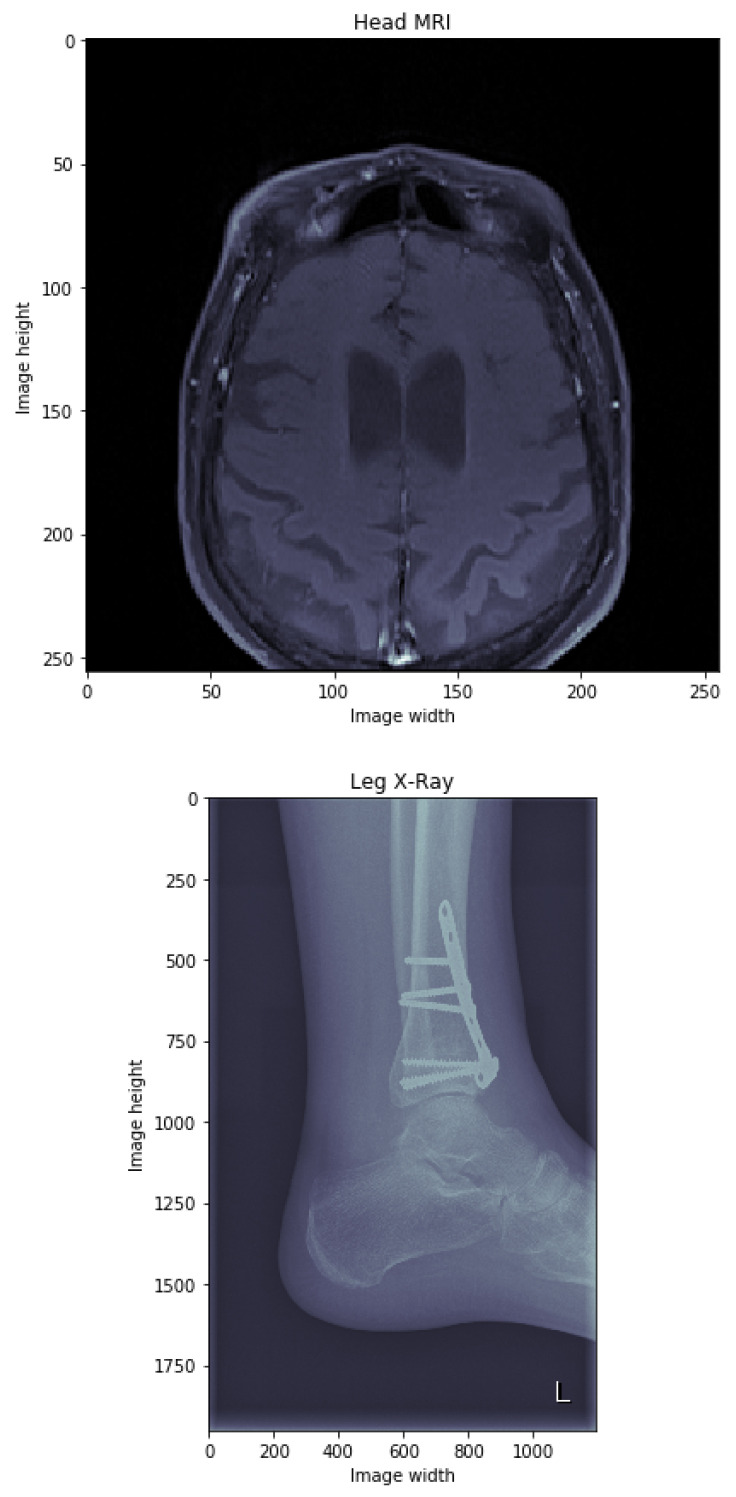
Example medical images. Original dimensions, expressed using the number of pixels, are indicated on each axis.

The first step in model training is the definition and training of the autoencoder, where the main goal is to use the encoder part as the core feature extractor, which will later be fine-tuned using the clustering module. The autoencoder is trained on images only, having the goal of reconstructing original medical images. The first problem that had to be addressed was related to the fact that medical images of different modalities can vary in size greatly, depending on the performed medical procedure. In Figure 2, two examples of medical images are shown, such that they greatly vary in dimensions—e.g., one slice of the head MRI is of dimensions 256×256, whereas the X-ray of the leg is 1952×1192. Because the resized smaller images (e.g., 28×28, 32×32, and so on) do not contain a sufficient level of detail which could make them easily visually distinguishable from one another, while larger images (e.g., 1024×1024 or 2048×2048) would result in overly complex models, further resulting in increased variation, we decided to resize all the images to dimensions 256×256. All pixel intensities were normalised to the interval [0,1]. Having in mind the scalability issues that would occur when using traditional dense architecture, we opted for using a CAE instead [26,27]. A CAE is a neural network trained in an unsupervised manner, having the goal of reconstructing the original image, and whose architecture consists of convolutional layers (instead of dense layers). It consists of the encoder part, which takes an input image and maps it to a latent space, and a decoder part which uses the latent space and tries to reconstruct the original image from it.

**Figure 3:**
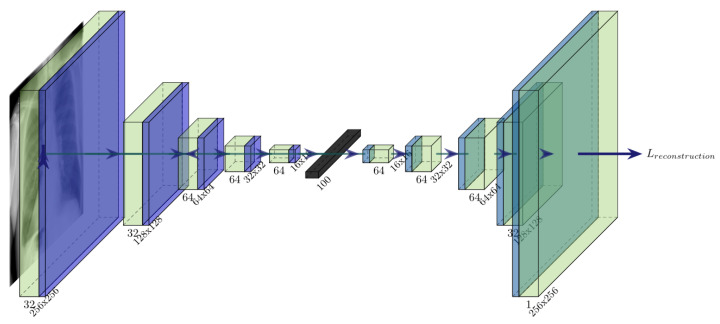
Architecture of the proposed convolutional autoencoder, which is later fine-tuned using pairwise constraints.

As can be seen in Figure 3, our autoencoder consists of the encoder part and a decoder part, both connected through a dense layer, which is later used as a feature extractor. During our tests, we tried to reduce the dimensionality of the dense layer as much as possible while preserving a small reconstruction error. Our tests have shown that a 100-dimensional dense layer attains satisfying reconstruction results while noticeably reducing the dimensionality from the previous layer. The encoder consists of five layers, where every layer consists of a 3×3 convolutional layer followed by a 2×2 max-pooling layer. Layers 1 and 2 have 32 convolutional filters each, whereas layers 3, 4, and 5 have 64 filters each. The decoder follows a symmetric five-layer layout, where each layer consists of a bilinear upsampling layer, followed by a convolutional layer. In contrast, using the transposed convolutional layer in architecture, the proposed decoder architecture avoids checkerboard patterns [28], thus having a better reconstruction error during training. All layers use the *ReLu* activation function (a=max(0,z)), except the last layer which utilises a *sigmoid* activation function ($a=\frac{1}{1+e^{-z}}\in \left[0,1\right]$). We use *mean-squared error* (MSE) as the loss function, and *Adam* as the optimiser, using the learning rate of 0.001. We train the model in batches of size 50. All hyperparameters, involving also model architecture, were defined purely by trial and error on validation data, using the values reported in related work for orientation. For example, our empirical tests suggest that increasing the number of filters (per layer) does not improve the model reconstruction error.

### 3.2. Using Gower Distance to Define Pairwise Constraints

Because DICOM tags consist of numerical as well as categorical data, using *Euclidean* or *cosine* distance as a method of estimating similarity between tags is not directly applicable. Therefore, we apply a distance measure proposed by *Gower* [29]. The similarity index is calculated using the following expression:

(1) $$S_{ij}=\sum_{k=1}^{p}s_{k}\left(x_{ik},x_{jk}\right)\delta_{k}\left(x_{ik},x_{jk}\right)/\sum_{k=1}^{p}\delta_{k}\left(x_{ik},x_{jk}\right),$$

where *p* is the total number of features, and $s_{k}$ is the similarity score between *k*-th feature of the data instances *i* and *j*. Because there exists a possibility that a specific feature is not observed in specific instances, δ factor is calculated in the following way: it equals 0 if the factors are not comparable, and is 1 otherwise. This solves the problem of missing values in the data.

For categorical features, the similarity score between the *k*-th categorical feature of data instances *i* and *j* is calculated using the expression:

(2) $$s_{k}\left(x_{ik},x_{jk}\right)=\left\{\begin{array}{cc} 1 & x_{ik}\;=\;x_{jk},\\ 0 & \mathrm{otherwise}, \end{array}\right.$$

and, for numerical features, the similarity score is calculated using the expression:

(3) $$s_{k}\left(x_{ik},x_{jk}\right)=\frac{|x_{ik}-x_{jk}|}{R_{k}},$$

where $R_{k}$ denotes the range for the *k*-th feature, i.e., $R_{k}=\max_{i}x_{ik}-\min_{i}x_{ik}$. Finally, the *Gower* distance between two data instances, *i* and *j*, is calculated using the following expression:

(4) $$\mathrm{Gower}\left(x_{i},x_{j}\right)=\sqrt{1-S_{ij}}.$$

Using the expression (4), we can calculate pairwise distances between the metainformation of instances, where it exists. Furthermore, it is reasonable to assume that similar images also have a lower *Gower* distance. By putting thresholds on the distance matrix, it is possible to calculate pairwise relations that can be utilised to improve the image clustering performance:

(5) $$m_{ij}=\left\{\begin{array}{cc} 1 & \mathrm{Gower}(x_{i},x_{j})<\epsilon ,\\ 0 & \mathrm{otherwise}, \end{array}\right.$$

(6) $$c_{ij}=\left\{\begin{array}{cc} 1 & \mathrm{Gower}(x_{i},x_{j})>\phi ,\\ 0 & \mathrm{otherwise}. \end{array}\right.$$

Following the expressions (5) and (6), it is important to note that there are three types of relations between pairs of instances. The first two are *must-link* and *cannot-link* relations. The third type of relation is *unknown*, and there are two reasons why it can occur. The first type of *unknown* relations can happen if during the comparison, the DICOM tags of at least one data instance are not known, whereas in the second case, *Gower* distance is neither high nor low, so we cannot be certain if the pair of data instances should fall in the same cluster. We delineate two square matrices for the labelled pairwise relations, *M* and *C*, which will be used for defining the additional pairwise loss:

(7) $$M=\left[\begin{array}{ccc} m_{11} & m_{12} & \cdots \\ \vdots & \ddots & \\ m_{N1} & \; & m_{NN} \end{array}\right],$$

(8) $$C=\left[\begin{array}{ccc} c_{11} & c_{12} & \cdots \\ \vdots & \ddots & \\ c_{N1} & \; & c_{NN} \end{array}\right].$$

Finally, we should note that the similarity $s_{k}$ can be additionally weighted. However, because there is no unique weighting solution that can improve the clustering performance [30] and it can sometimes even aggravate the clustering performance [31], we weigh all the variables uniformly.

Next, we describe a semi-supervised algorithm that utilises pairwise relations, as well as images, for training the cluster-oriented embeddings.

### 3.3. Semi-Supervised Clustering with Pairwise Constraints

When it comes to data analysis, pattern matching or machine learning, clustering is a task of grouping similar data instances based on some predefined similarity measure. In the case where neural networks are used to perform clustering, the loss functions of almost all reported algorithms have the common goal of minimising the weighted sum of the clustering loss ($L_{clustering}$) and the reconstruction loss ($L_{reconstruction}$):

(9) $$L_{unsupervised}=\alpha L_{clustering}+\beta L_{reconstruction},\;\alpha ,\beta \geq 0.$$

One of the most popular algorithms from this family of algorithms is the deep embedded clustering (DEC) algorithm [18]. The main idea of DEC is to minimise clustering loss which is defined as the *Kullback–Leibler* divergence (KL) between the soft assignments *q*, and an auxiliary distribution *p*, as is shown in:

(10) $$L_{clustering}=\mathrm{KL}\left(P||Q\right)=\sum_{i}^{N}\sum_{j}^{K}p_{ij}\log\frac{p_{ij}}{q_{ij}},$$

where *N* is the number of data points, and *K* is the predefined number of clusters. Soft assignment $q_{i}$ is the similarity between the embedding $z_{i}$ and the cluster centre $\mu_{j}$, which is calculated using Student’s *t*-distribution:

(11) $$q_{ij}=\frac{{\left(1+{∥z_{i}-\mu_{j}∥}^{2}\right)}^{-1}}{\sum_{j^{\prime }}{\left(1+{∥z_{i}-\mu_{j^{\prime }}∥}^{2}\right)}^{-1}},$$

while the auxiliary distribution is calculated using the *p* distribution:

(12) $$p_{ij}=\frac{q_{ij}^{2}/\sum_{i}q_{ij}}{\sum_{j^{\prime }}q_{ij^{\prime 2}}/\sum_{i}q_{ij^{\prime }}}.$$

To improve the clustering results even further, Guo et al. [32] proposed the *improved DEC* (IDEC) model, where MSE is added to the original DEC loss. In this equation, $x_{i}$ is the *i*-th datapoint, while ${\hat{x}}_{i}$ is the decoder output of the *i*-th datapoint:

(13) $$L_{reconstruction}=\sum_{i=1}^{n}{∥x_{i}-{\hat{x}}_{i}∥}_{2}^{2}.$$

Although the DEC and IDEC algorithms can achieve state-of-the-art results for some datasets, such as MNIST [33] or REUTERS-10k [34], when it comes to clustering medical images, they fail to attain noticeably better clustering results compared to the standard clustering algorithms, such as k-means. However, their performance can be improved by adding additional information, along with image data, that can be used to construct pairwise constraints.

We propose adding a pairwise loss which is similar to the loss defined in [20], where the main goal is to decrease the KL divergence between soft cluster assignment distributions for pairs of instances that should belong to the same cluster and increase it for pairs of instances that should fall into different clusters, defined by:

(14) $$L_{pairwise}=\frac{1}{n}\sum_{i=1}^{n}\sum_{j=1}^{n}m_{ij}\mathrm{KL}(q_{j}\left|\right|q_{i})+\frac{1}{n}\sum_{i=1}^{n}\sum_{j=1}^{n}c_{ij}\max(0,\mathrm{margin}-\mathrm{KL}(q_{j}\left|\right|q_{i})).$$

The main reason for choosing such a loss function instead of a regular, contrastive loss [35] with *Euclidean* distance lies in the fact that the chosen loss function cannot be directly applied to $q_{i}$ because it is a probability distribution, and even if it were applied directly, the embeddings $z_{i}$ would not have any impact in optimising the cluster centroids μ for the data where pairwise constraints are known.

Combining the Equations (9) and (14) with the reconstruction loss, we get the following loss function:

(15) $$L=\alpha L_{clustering}+\beta L_{reconstuction}+\gamma L_{pairwise}$$

**Figure 4:**
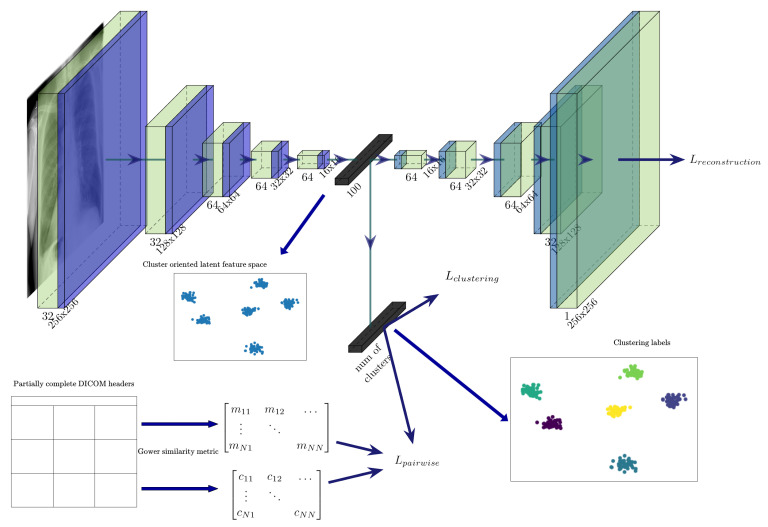
The architecture of the proposed semi-supervised algorithm for learning cluster-oriented representations.

The architecture of the proposed model is illustrated in Figure 4. As it can be seen in the figure, the fully-connected layer that is connecting the encoder with the decoder part of the neural network is later used as a feature extractor, whereas the clustering layer, which is also connected to the already mentioned layer, is used to generate a probability distribution for an instance, assigning it to a specific cluster.

Minimisation of the loss function *L* is done using stochastic gradient descent and back-propagation. During back-propagation, we update the encoder weights $W_{e}$, decoder weights $W_{d}$, as well as the cluster centres $\mu_{i}$. The updates are made using the following expressions:

(16) $$\mu_{j}=\mu_{j}-\frac{\lambda }{m}\sum_{i=1}^{m}\left(\alpha \frac{\partial L_{clustering}}{\partial \mu_{j}}+\gamma \frac{\partial L_{pairwise}}{\partial \mu_{j}}\right),$$

(17) $$W_{e}=W_{e}-\frac{\lambda }{m}\sum_{i=1}^{m}\left(\alpha \frac{\partial L_{clustering}}{\partial W_{e}}+\beta \frac{\partial L_{reconstruction}}{\partial W_{e}}+\gamma \frac{\partial L_{pairwise}}{\partial W_{e}}\right),$$

(18) $$W_{d}=W_{d}-\frac{\lambda }{m}\sum_{i=1}^{m}\left(\beta \frac{\partial L_{reconstruction}}{\partial W_{d}}\right),$$

where λ is the learning rate, and *m* is the number of data instances in a mini-batch. To calculate the encoder weights $W_{e}$, firstly the gradients $\partial L/\partial z_{i}$ are calculated and are then passed down to the network to calculate the $\partial L/\partial W_{e}$.

To better illustrate how model training is performed, detailed pseudocode is shown in Algorithm 1.

**Algorithm 1** Semi-supervised model-training algorithm utilising DICOM tags and images**Require:**   Dataset ${\left\{x\right\}}_{i=1}^{n}$ (images coupled with DICOM tags, where available), number of clusters *K*, weights for the loss function (α, β, γ), ϵ and ϕ for *Gower* distance used to calculate *must-link* and *cannot-link* pairwise relations, tol threshold for stopping the training, batch_size, margin.1:Train CAE on images, only to obtain the initial image embeddings ${\left\{z_{i}\right\}}_{i=1}^{n}$2:Perform k-means on the latent space *Z* to obtain the initial cluster estimation, as well as the cluster centres3:Calculate pairwise relations using *Gower* distance, considering the thresholds ϵ and ϕ4:**for** epoch∈{0,1,...,num_epochs} **do**5:    **if** epoch%update_interval==0 **then**6:        Compute $p_{ij}$ according to Equation (12)7:        Save old clustering assignments $c_{old}⇐{\left\{c\right\}}_{i=1}^{n}$8:        Update clustering assignments $c_{i}⇐\underset{j}{\mathrm{argmax}}q_{i}$9:        **if** $(\sum_{i}^{n}c_{old}\neq c)/tol$ **then**10:           stop training11:        **end if**12:    **end if**13:    **for** mini_batch∈{0,1,...,num_mini_batches} **do**14:        Update network parameters θ, as well as the cluster centres ${\left\{\mu \right\}}_{i=1}^{K}$ according to Equations (17)–(18)15:    **end for**16:**end for**


Because the matrices *M* and *C* are sparse, and the largest possible number of pairwise constraints inside the dataset can be n(n−1)/2, where *n* is the number of data instances in the training set, the probability of two instances having a pairwise constraint falling in the same mini-batch is rather small when using uniform random sampling. Therefore, to compensate, we implemented our batch sampler to make sure that every mini-batch contains at least one *must-link* and one *cannot-link* constraint. We implemented our model using the PyTorch framework [36]. Our experiments were performed on a computer consisting of two Intel^®^ Xeon^®^ Processors E5-2620 v4 CPUs, 128 GB of RAM and having three GeForce RTX 2080 Ti graphic cards. Although even one graphics card was sufficient for training the model, we used all three cards simultaneously to train multiple models in parallel, which shortened the time to find the most promising hyperparameter values (Section 3.5).

### 3.4. Dataset

To demonstrate the performance of the proposed semi-supervised method in which we used a small amount of supervised data to construct pairwise relations, we use a clinical dataset originating from the Clinical Hospital Centre (CHC) Rijeka. The original dataset consists of approximately 30 million images of regular exams (images and DICOM tags), acquired through standard clinical practice at the CHC Rijeka, between 2010 and 2017. From these, approximately 14 million images contain at least one DICOM tag. Because the data stored in the relational database was not informative enough to have any relevance for image clustering, we only analysed the DICOM tags associated directly with specific images.

Images were retrieved and stored on a GPU workstation in the possession of the Faculty of Engineering in Rijeka (RITEH), along with additional information from the relational database, connecting the images to specific exams. Because querying and retrieving the needed information (images and/or DICOM data) from the file system was computationally challenging, the first step we made was to separate the DICOM tags and store them in a separate database, one which would be more manageable. This resulted in a 40 GB database that we could load into the workstation RAM, which in turn enabled us to perform any kind of descriptive analysis much faster.

Furthermore, because training the models on the entire collection of images could require days, we randomly sampled two disjoint data subsets reflecting the distribution of the DICOM tags in the whole dataset: a training subset consisting of 30,000 images, and a test subset consisting of 8000 images. Because there are approximately 4000 possible DICOM tags, and most of them are not present even once in our dataset, we chose to use only the following tags: *Modality* (Mod), *BodyPartExamined* (BPE), *PatientPosition*, *MRAcquisitionType*, *ImageOrientationPatient*, *Manufacturer*, *ExposureTime*, and *Exposure*. These tags were selected because they introduce basic information that is required to differentiate between two medical images and are explainable even without consulting the radiology experts. All tags except *ImagePositionPatient*, *ExposureTime*, and *Exposure* are categorical. The tag *ImagePositionPatient* consists of 6 values representing two normalised three-dimensional vectors that are used to describe the orientation of the patient with respect to the reference coordinate system. We should note that the *Mod* tag is fully present in both the training and the test set. This can be explained by the fact that it is filled in automatically by the device that performs the imaging procedure. However, *BPE* is only partially available—it is missing mainly for the X-ray imaging modality. During our analysis, we noticed that the *StudyDescription* tag, which is edited manually by a physician and is relatively short in size per record, can be used in combination with the *BPE* tag to reconstruct more accurate information concerning the examined anatomical region (*AR*), often missing in the *BPE* tag. By searching the keywords concerning the *BPE* tag from the DICOM documentation (http://dicom.nema.org/medical/dicom/current/output/chtml/part16/chapter_L.html##chapter_L (last accessed on 1 October 2021)), we were able to reconstruct all the missing information about the examined anatomical regions. However, because we wanted to test how our algorithm performs on raw DICOM tags, we used this extracted information only during the validation process.

Concerning images, our dataset consists of the following imaging modalities: CT (computed tomography), XA (X-ray angiography), NM (nuclear medicine), RF (radio fluoroscopy), MR (magnetic resonance), and CR (computed radiography). All used images are two-dimensional; slices composing 3D modalities were treated as independent. There are 23 different *AR* labels in the dataset. Although the modalities are equally distributed in the dataset, the same does not hold for the *AR* labels.

### 3.5. Model Evaluation and Experimental Setup

When it comes to the performance analysis of the proposed method, several factors need to be taken into consideration. Firstly, it is necessary to investigate how specific hyperparameters affect clustering performance. These include the number of clusters *K* and the weights for the clustering loss function itself, where we balance between preferring labelled or unlabelled data. Next, when reasonably good hyperparameter values were found, we tested and compared our model to the deep unsupervised CAE and IDEC models, as well as other, standard clustering algorithms such as unsupervised k-means, semi-supervised COP k-means, and PC k-means. Furthermore, to visualise the embeddings in the two-dimensional space, we applied *t-SNE* [37] to the test dataset on CAE, IDEC, and the proposed model. Finally, we tested how the proposed algorithm behaves concerning the ratio of unlabelled and labelled data inside the training data.

First, we searched for an adequate value of the number of clusters and the hyperparameter values shaping the loss function, with regard to the NMI score. We inspected the model performance for the following values of the number of clusters K={5,10,15,20,25,30,35}. Furthermore, because there are three weights that can be adjusted in the loss function (α, β, γ), repeated training of multiple combinations of hyperparameter values would be time-consuming. To reduce the number of hyperparameters under consideration, we chose α=0.1 and β=1, as already used in [32,38]. We performed the search using the following values of γ={0,0.1,1,10,100}, which indicates the level of importance assigned to labelled data. Values and ranges of γ and *K* were selected intuitively. Same as for unsupervised CAE pretraining, *Adam* was chosen as the optimiser, using a learning rate of 0.001 and the mini-batch size of 50. *Margin* from the Equation (14) was set to be 1; we also tested model performance using other margin values (e.g., 2); however, this did not result in a noticeable improvement. Each training was performed through 100 full-batch epochs. Moreover, because we noticed that the results depend on the initial k-means estimation of the clusters, for every combination of the training parameters we repeated our training procedure 10 times and considered the mean values for choosing the optimal values.

After establishing the solid values of *K* and γ, we explored how the proposed model behaves on different sizes of pairwise constraints sets, as well as the ratio of labelled data instances from which the pairwise constraints can be sampled.

During the test, we utilised several validation methods to track the algorithm performance. To monitor the cluster structure, we used *silhouette score* [39]. *Silhouette score* is an internal evaluation method that shows how well the data points are clustered, taking into consideration cluster tightness and the separation between clusters. It is calculated using the following expression:

(19) $$s\left(i\right)=\frac{b(i)-a(i)}{\max\{a(i),b(i)\}},$$

where a(i) is the mean distance between *i*-th instance and all other instances falling into the same cluster, and b(i) is the smallest mean distance from *i*-th instance to all the instances not falling into the same cluster:

(20) $$a\left(i\right)=\frac{1}{|C_{i}|-1}\sum_{j\in C_{i},i\neq j}d\left(i,j\right),$$

(21) $$b\left(i\right)=\min_{k\neq i}\frac{1}{|C_{k}|}\sum_{j\in C_{k}}d\left(i,j\right),$$

where d(i,j) is the distance between the instances *i* and *j*, and $|C_{i}|$ is the number of instances falling into cluster *i*.

Although the *silhouette score* is a good method for assessing cluster structure, it still does not tell us anything about the semantic structure of the elements inside the clusters. Therefore, we also used the *normalised mutual information* (NMI) and the *homogeneity score* (HS) [40] as external measures to verify if the elements inside the clusters are semantically similar. NMI is calculated using the following expression:

(22) $$NMI\left(y,c\right)=\frac{I(y,c)}{\frac{1}{2}\left[H\left(y\right)+H\left(c\right)\right]},$$

where *y* represents ground truth labels, *c* represents cluster labels, I(y,c) represents the mutual information, and *H* is the entropy. Finally, we used HS, falling in the range [0,1], for showing the homogeneity of labels falling in specific clusters: 1 tied to perfectly homogeneous clusters and 0 tied to completely random clusters are present inside the specific cluster, the score being calculated using the following expression:

(23) $$HS\left(y,c\right)=1-\frac{H(C|K)}{H(C)},$$

where H(c|k) is calculated using the expressions:

(24) $$H\left(C|K\right)=-\sum_{k=1}^{\left|K\right|}\sum_{c=1}^{\left|C\right|}\frac{a_{ck}}{N}log\frac{a_{ck}}{\sum_{c=1}^{\left|C\right|}a_{ck}},$$

(25) $$H\left(C\right)=-\sum_{c=1}^{\left|C\right|}\frac{\sum_{k=1}^{\left|K\right|}a_{ck}}{n}log\frac{\sum_{k=1}^{\left|K\right|}a_{ck}}{n}.$$

In Equations (23)–(25), *K* is the number of clusters, *C* is the number of labels and $a_{ck}$ is the number of data instances belonging to the *k*-th cluster while being of class *c*.

Although the HS cannot be used for the evaluation and comparison of the clustering results by itself, if it is combined with other methods, it can be useful for additionally analysing the clustering results. Moreover, it is important to note that in our specific case, occurrences of similar data instances scattered across multiple clusters, i.e., multiple clusters delineating the same label, were not regarded as detrimental.

To test the clustering performance, we chose the information concerning the anatomical region from the *StudyDescription* tag and the *Mod* tag as class labels that will be used in calculating NMI and HS. We decided not to use other categorical tags for model evaluation because their domains (i.e., their ranges of possible unique values) are much smaller and are hence easier to cluster.

## 4. Results

As described in Section 3, we performed two experiments to find optimal parameters *K* and γ by observing how they affect the NMI of the already mentioned DICOM tags. These experiments were performed using 2000 pairwise relations. Furthermore, when generating the pairwise relations, we defined that only the pairs having the *Gower* distance of 0.1 or lower are considered to be *must-link* (i.e., ϵ<0.1), whereas *cannot-link* pairs are calculated if two data instances have a *Gower* distance higher than 0.5 (i.e., ϕ>0.5). In the first experiment, using trial and error, we observed that the model performs well using the parameter γ=10. Using this value of γ, we ran the first experiment to observe how the number of clusters will affect the clustering performance. In the second experiment, we observed for a specific number of clusters K=25 how the model performs by varying the value of γ.

In Table 1, clustering performance with respect to the number of clusters *K* given γ=10 is shown. As can be observed in the table, clustering performance for the *AR* is increasing up to the size of 10 clusters; further increases in the number of clusters fail to make a difference in terms of the applied evaluation methods. Furthermore, the results suggest that the model having 25 clusters achieved the best performance in the clustering of the *AR*.

**Figure 5:**
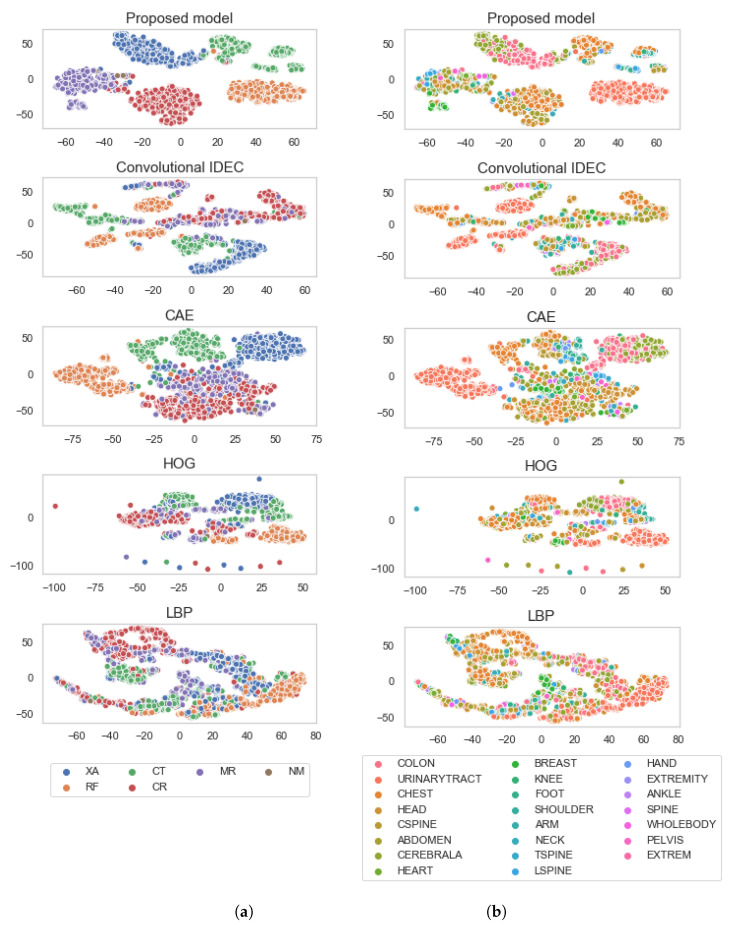
t-SNE visualisation of the embedding space. Each row depicts the embedding space of one of the modelling approaches used in the experiments, with respect to the: (**a**) *Mod* tag, and (**b**) *AR* information.

In Table 2, we show how model performance changes with respect to the value of γ, using K=25 clusters. During this test, we also noticed that the standard unsupervised loss has a greater impact on increasing the *silhouette score*, whereas adding more weight to the pairwise loss (increasing the value γ) increases NMI and HS. As can be seen in Table 2, for γ=10, we achieved the best clustering result on *AR*, whereas for γ=100, we get the best clustering result on the *Mod* tag. However, utilising such high γ values fails to reflect positively on the *silhouette score*, which indicates that the clustering structure is weaker, meaning that either the different clusters are closer to one another, or the instances inside a specific cluster are more distant from each other. Therefore, we can conclude that the unsupervised loss ensures that the clusters are tight and well separated; however, it does not ensure that the data inside the clusters will be semantically similar. On the other hand, the pairwise loss has an impact on making the clusters more semantically similar. Additionally, to visualise the embedding space of the test set, we used t-SNE to reduce the dimensionality of the embedding space into two dimensions. The visualisations are shown in Figure 5. We can observe that the proposed model results in greater-sized clusters and a more homogeneous embedding space compared to the remaining feature descriptors. One such example can be seen when comparing the embedding space of the proposed model with the CAE where the proposed model better separates *CT* and *MR* modalities.

Next, we compare our model against several unsupervised and supervised learning algorithms, combined with several feature descriptors. Both a histogram of oriented gradients (HOG) and local binary pattern (LBP) were selected as commonly used feature descriptors in the analysis of medical images [41,42]. CAE was selected as the first stage in algorithm training to observe how the algorithm performance changes in different training phases. Both DEC and IDEC were selected as an unsupervised predecessor of the proposed algorithm. For HOG, 8×8 cells with 2×2 cells per block were selected as parameters, while for LBP the radius was set to 1, the number of neighbouring points was set to 8, and 16×16 cells were used. Constrained k-means (COP k-means) [11,12] and pairwise constrained k-means (PC k-means) [13] were selected as semi-supervised clustering algorithms to enhance the clustering performance of the previously described feature descriptors. Table 3 suggests that our model noticeably outperforms all other models. We should note that the unsupervised convolutional IDEC was trained with the same α and β hyperparameter values, as well as the same initial CAE weights, and using the initial cluster assignments. We should also note that the convolutional IDEC also outperforms both k-means and the semi-supervised COP k-means and PC k-means with respect to the *Mod* tag.

**Figure 6:**
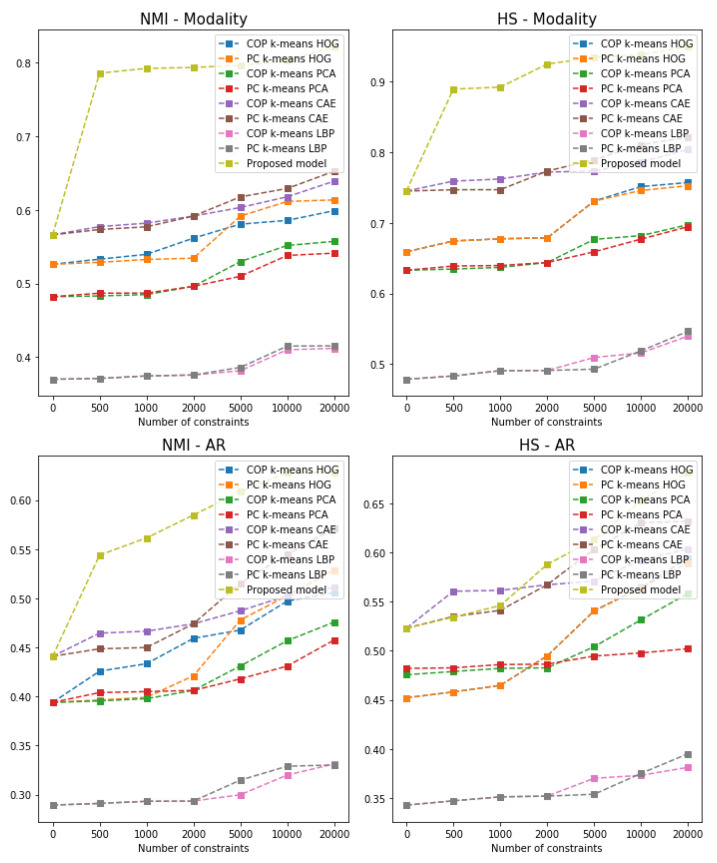
Clustering results on the train and test subsets with respect to the percentage of labelled instances used and the number of constraints introduced.

To analyse how the proposed model behaves on different sizes of pairwise constraints sets, as well as the ratio of supervised data instances from which the pairwise constraints can be sampled, we tested our model on 500, 1000, 2000, 5000, 10,000, and 20,000 constraints. The results are shown in Figure 6. We can observe that having only 500 pairwise constraints brings a noticeable improvement in the clustering results. Moreover, as the number of pairwise constraints increases, the number of instances from which the data can be sampled has a greater impact on increasing the clustering performance. It is important to note that for very small numbers of pairwise constraints (e.g., less than 2000), COP k-means coupled with CAE shows better performance at clustering the *AR*.

## 5. Discussion

In this paper, we propose an algorithm for semi-supervised clustering of medical images using both images as well as (partially complete) DICOM tag metadata from a fraction of the available data.

We show that DICOM data can be used to generate pairwise constraints that can help increase the clustering performance of medical images, even when using only a small number of constraints (e.g., 500 constraints). We can conclude that the proposed model architecture can generalise well, as we demonstrated by evaluating model performance on test data using several evaluation methods. We also confirm by visual inspection that it groups visually similar images, even when having only partially observable DICOM metainformation.

We also show that the algorithm performs worse for *AR* in comparison with *Mod*. Because different *AR*s inside a single modality are much more similar to one another, compared to images of different modalities, we hypothesise that the existing DICOM tags could be enriched with additional tags or with some additional source of information (e.g., textual diagnosis) in the future to increase the clustering accuracy. Moreover, data imbalance is also not taken into consideration, which could result with minority class data points not being clustered together, especially if there are insufficient pairwise constraints that define relations for such instances. Finally, with the increase of images containing DICOM tags, the number of pairwise constraints grows quadratically, increasing resource requirements for the training environment. This problem could be reduced by using special structures for sparse matrices or by defining criteria for selecting only specific pairwise constraints and removing the trivial ones.

There are several possible practical applications of the proposed model. Firstly, it could be used as a foundation for building CBMIR systems, which can help both medical professionals, as well as computer scientists, to perform various data mining tasks on large repositories of medical images that are extracted from PACSs. In addition, it could be used to impute the missing metadata or fix erroneous DICOM tags by leveraging the clustering labels, which are the model output together with the existing DICOM tags. Finally, it is important to note that all the applications mentioned above can be done using the proposed model with only a fraction of partially-labelled data (e.g., 2500 labelled out of the 30,000 instances total used for model training).

Although the results presented in our study look promising, we believe that model performance can be further improved by exploring several future research directions. First, alternative network architectures, such as generative adversarial networks (GANs) [43] or variational autoencoders (VAEs) [44], should be explored, which would require designing suitable approaches for incorporating the information contained in the DICOM tags into these models. Furthermore, it might be beneficial to experiment with alternative cost functions, especially with the part of the cost function that utilises pairwise distances, e.g., using the classical contrastive loss by optimising the *Euclidean* distance. Additionally, possible improvements could be achieved by examining different DICOM tags weighting strategies. Finally, it would be interesting to evaluate model performance to fill in the missing DICOM tag values, as well as detecting errors in the observed DICOM tag values.

## Figures and Tables

**Table 1:** Clustering performance obtained for varying numbers of clusters *K* on train/test data with respect to the two selected labels (*Mod* and *AR*), using the following loss-function weights: α=0.1, β=1 and γ=10. The best result in each column is printed in boldface. *Silhouette score* is not shown in this table because it does not depend on the number of clusters.

Number of Clusters	Train NMI AR	Train HS AR	Train NMI Mod	Train HS Mod	Test NMI AR	Test HS AR	Test NMI Mod	Test HS Mod
5	0.397	0.317	0.743	0.702	0.386	0.308	0.744	0.703
10	0.518	0.516	**0.826**	0.887	0.516	0.504	**0.828**	0.890
15	0.525	0.516	0.811	**0.910**	0.529	0.510	0.805	0.904
20	0.545	0.536	0.797	0.898	0.541	0.533	0.792	0.898
25	**0.565**	**0.546**	0.793	0.911	**0.584**	**0.587**	0.793	0.911
30	0.511	0.514	0.782	0.897	0.505	0.508	0.778	0.892
35	0.544	0.537	0.754	0.914	0.528	0.527	0.752	**0.913**

**Table 2:** Performance of the proposed model with respect to the value of γ, where K=25 clusters. The remaining loss-function weights are fixed to the following values: α=0.1 and β=1. The best result in each column is printed in boldface.

γ	Silhouette Score	Train NMI AR	Train HS AR	Train NMI Mod	Train HS Mod	Test NMI AR	Test HS AR	Test NMI Mod	Test HS Mod
0	**0.726**	0.487	0.533	0.636	0.823	0.473	0.544	0.637	0.755
0.1	0.715	0.496	0.545	0.656	0.834	0.479	0.525	0.657	0.843
1	0.650	0.516	0.554	0.679	0.867	0.501	0.539	0.674	0.861
10	0.638	**0.586**	**0.563**	0.799	0.912	**0.584**	**0.587**	0.793	0.911
100	0.350	0.543	0.545	**0.806**	**0.917**	0.536	0.531	**0.801**	**0.913**

**Table 3:** Performance of the proposed model against unsupervised k-means, unsupervised convolutional IDEC, semi-supervised COP k-means, and PC k-means, using several feature descriptors. For semi-supervised models, 2000 constraints were used. The proposed model is trained using the following hyperparameter values: α=0.1, β=1 and γ=10. The results shown represent the mean obtained from 10 independent iteration runs. Best results are emphasised.

Feature Descriptor	Algorithm	Test NMI AR	Test HS AR	Test NMI Modality	Test HS Modality
PCA	K-means	0.394	0.342	0.482	0.633
	COP K-means	0.405	0.473	0.496	0.643
	PC K-means	0.406	0.486	0.496	0.645
CAE	K-means	0.441	0.523	0.566	0.745
	COP K-means	0.463	0.545	0.581	0.771
	PC K-means	0.449	0.541	0.576	0.773
HOG	K-means	0.394	0.451	0.526	0.659
	COP K-means	0.433	0.452	0.561	0.677
	PC K-means	0.409	0.464	0.534	0.673
LBP	K-means	0.289	0.291	0.369	0.478
	COP K-means	0.293	0.351	0.374	0.490
	PC K-means	0.299	0.356	0.371	0.491
Convolutional IDEC		0.473	0.544	0.637	0.755
Proposed model		**0.584**	**0.587**	**0.793**	**0.911**

## Abbreviations

The following abbreviations are used in this manuscript:

PACS	Picture Archiving and Communication System
DICOM	Digital Imaging and Communications in Medicine
CAE	Convolutional autoencoder
MSE	Mean squared error
DEC	Deep embedded clustering
IDEC	Improved deep embedded clustering
PC k-means	Pairwise constrained k-means
KL	Kullback–Leibler
NMI	Normalised mutual information
HS	Homogeneity score
PCA	Principal component analysis
HOG	Histogram of oriented gradients
LBP	Local binary pattern
CHC	Clinical Hospital Centre
Mod	Modality
BPE	Body Part Examined
AR	Anatomic region
CT	Computed tomography
XA	X-ray angiography
NM	Nuclear medicine
RF	Radio fluoroscopy
MR	Magnetic resonance
CR	Computed radiography
